# Comparison of Methods for Estimating Retinal Shape: Peripheral Refraction vs. Optical Coherence Tomography

**DOI:** 10.3390/jcm10020174

**Published:** 2021-01-06

**Authors:** Katharina Breher, Alejandro Calabuig, Laura Kühlewein, Focke Ziemssen, Arne Ohlendorf, Siegfried Wahl

**Affiliations:** 1 Institute for Ophthalmic Research, University of Tübingen, 72076 Tübingen, Germany; alejandro.calabuig-barroso@uni-tuebingen.de (A.C.); laura.kuehlewein@med.uni-tuebingen.de (L.K.); siegfried.wahl@uni-tuebingen.de (S.W.); 2 Center for Ophthalmology, University of Tübingen, 72076 Tübingen, Germany; Focke.Ziemssen@med.uni-tuebingen.de; 3 Carl Zeiss Vision International GmbH, 73430 Aalen, Germany; arne.ohlendorf@medizin.uni-tuebingen.de

**Keywords:** myopia, retinal shape, peripheral refraction

## Abstract

Retinal shape presents a clinical parameter of interest for myopia, and has commonly been inferred indirectly from peripheral refraction (PRX) profiles. Distortion-corrected optical coherence tomography (OCT) scans offer a new and direct possibility for retinal shape estimation. The current study compared retinal curvatures derived from OCT scans vs. PRX measurements in three refractive profiles (0${}^{{}^{\circ}}$ and 90${}^{{}^{\circ}}$ meridians, plus spherical equivalent) for 25 participants via Bland–Altman analysis. The radial differences between both procedures were correlated to axial length using Pearson correlation. In general, PRX- and OCT-based retinal radii showed low correlation (all intraclass correlation coefficients < 0.21). PRX found flatter retinal curvatures compared to OCT, with the highest absolute agreement found with the 90${}^{{}^{\circ}}$ meridian (mean difference +0.08 mm) and lowest in the 0${}^{{}^{\circ}}$ meridian (mean difference +0.89 mm). Moreover, a negative relation between axial length and the agreement of both methods was detected especially in the 90${}^{{}^{\circ}}$ meridian (R = −0.38, *p* = 0.06). PRX measurements tend to underestimate the retinal radius with increasing myopia when compared to OCT measurements. Therefore, future conclusions from PRX on retinal shape should be made cautiously. Rather, faster and more clinically feasible OCT imaging should be performed for this purpose.

## 1. Introduction

Myopia is a spherical refractive error that commonly results from a mismatch between the focal and axial length of the eye. As the eye undergoes excessive growth, the risks for sight-threatening ocular pathologies, such as myopic macular degeneration and retinal detachment, are strikingly enhanced in myopes [1]. Due to various factors (see the review in [2]), its prevalence is estimated to increase, affecting up to half of the global population by the year 2050 [3] and leading to a public-health and socioeconomic burden [4].

Peripheral refraction (PRX) was reported to play a substantial role in myopia development [5]. In monkeys, for example, the induction of peripheral hyperopic defocus—even in combination with foveal ablation—resulted in central axial elongation [6]. Additionally, it was found in humans that uncorrected myopes show more relative peripheral hyperopia than emmetropes and hyperopes [7,8,9,10,11]. However, from longitudinal studies, it has been suggested that relative peripheral hyperopia is a consequence rather than the cause of myopia [12,13,14]. Moreover, the basis of retinal shape is interwoven with PRX and has commonly been concluded from PRX profiles [15]. The more relative the peripheral hyperopia exhibited is, the steeper and the more prolate the retina is suggested to be, as in the case of myopia [16,17,18,19]. However, it has already been stated that conclusions from PRX about retinal shape should be made cautiously, especially with respect to the high variability of off-axis ocular optics [15]. Nowadays, retinal shape can also be estimated using optical coherence tomography (OCT) in a clinically feasible manner. Here, the myopic retina was found to grow in a balloon-like fashion across the horizontal scan field [20], which partly stands in conflict with the previously predicted steepening of the retina as indirectly derived from PRX.

To the best of the authors’ knowledge, there have not yet been any studies conducted in order to analyze the agreement between the direct OCT-based retinal shape versus the indirect PRX-based retinal shape. Therefore, the present study will investigate the agreement of OCT- and PRX-based retinal curvature. It will evaluate whether a direct transfer from PRX to retinal shape can be recommended or if these two parameters should be considered separately. These findings are of interest for clinical environments when choosing the preferred procedure for retinal shape estimation for individual myopia progression follow ups, but also for individualized myopia control choices and efficacy evaluations.

## 2. Materials and Methods

### 2.1. Study Participants

This prospective, monocentric, and cross-sectional study was carried out at the Institute for Ophthalmic Research in Tübingen, Germany. It followed the Declaration of Helsinki and data protection regulations, and was approved by the ethics committee of the Medical Faculty of the University of Tübingen. Informed consent was obtained from every participant prior to the study measurements. Participants with ocular pathologies, previous ocular surgery, and insufficient OCT signal strength < 6—and, thus, reduced image quality of the OCT scans—were excluded. A total of *n* = 25 participants with a mean age of 24.6 ± 4.0 years (range 19 to 35 years) were included in the study. Measurements were performed on undilated right eyes. The mean axial length (ZEISS IOLMaster 700, Carl Zeiss Meditec AG, Jena, Germany) was 23.89 ± 0.75 mm (range 22.59 to 25.17 mm). The mean central refractive error (i.Profiler plus, ZEISS Vision, Aalen, Germany) was −1.07 ± 1.60 D (range −5.14 to +0.57 D). The group consisted of 14 emmetropes and 11 myopes.

### 2.2. OCT-Based Retinal Shape Estimation

**Figure 1:**
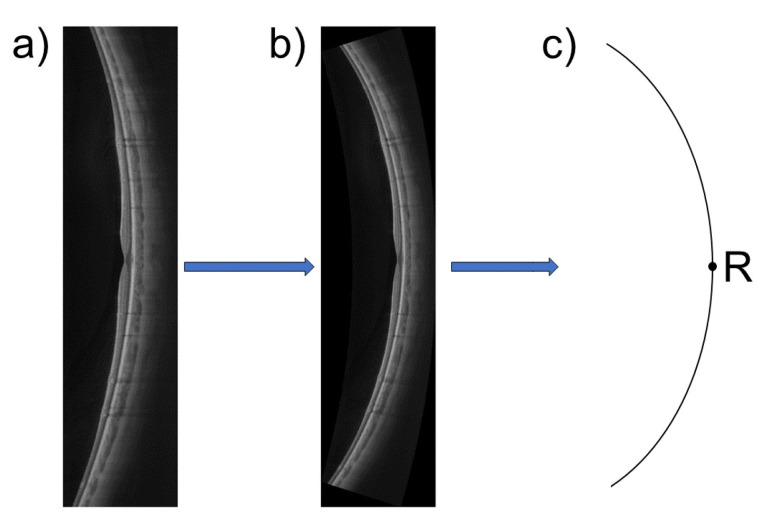
Process of retinal shape calculation from optical coherence tomography (OCT) imaging: (**a**) the original OCT scan; (**b**) the distortion-corrected OCT scan; (**c**) a circle is fitted to the retinal pigment epithelium (RPE) of the corrected scan image, and its radius is extracted for retinal shape estimation. The point “R” on this circle scheme is displayed for better reference to Figure 2.

Swept-source OCT imaging (ZEISS PlexElite 9000, Carl Zeiss Meditec Inc., Dublin, USA) was performed for retinal imaging with subsequent retinal shape calculation. The OCT scan pattern consisted of a horizontal 16 mm (approximately 52${}^{{}^{\circ}}$) line scan, which was centered on the fovea. In order to estimate the retinal shape, the OCT scan first had to be corrected for the scan geometry distortions. These geometrical distortions arise from the artificial flattening of the OCT image due to display reasons. A newly developed and validated optical model by Steidle and Straub [21] was used to perform the geometrical distortion correction. This was achieved via ray-tracing of the OCT A-scans through the optical components of the OCT device and the Arizona eye model [22] with the individually adjusted axial length of the participant using MATLAB (MATLAB 2018b, The MathWorks, Inc., Natick, MA, USA) and OpticStudio (OpticStudio, Zemax, LLC, Kirkland, WA, USA). After re-construction of the geometrically correct scan, the retinal radius of curvature was extracted from a circle that was fitted to the retinal pigment epithelium (RPE) of the OCT image (see Figure 1). This will be stated as the “OCT-based retinal curvature” hereinafter.

### 2.3. PRX-Based Retinal Shape Estimation

PRX measurements were performed using eccentric photorefraction as described elsewhere [23], but with the horizontal meridian being measured additionally in the same manner. The photorefractor consisted of a moving mirror to scan from the temporal to the nasal retina across a $\pm 50^{{}^{\circ}}$ scan field (measurement angle α). The sampling rate was $0.71^{{}^{\circ}}$. The fixation target was mounted in front of the right eye, such that profiles were measured in the primary gaze direction of the participants and were centered on the fovea. The photorefractor measures the vertical and horizontal profiles of refraction after each other—the $0^{{}^{\circ}}$ and $90^{{}^{\circ}}$ refractive meridians. The spherical equivalent profile was calculated as the average of the $0^{{}^{\circ}}$ and $90^{{}^{\circ}}$ meridian values. One measurement cycle was repeated from 7 to 14 times for each participant, depending on the compliance. With this repetitive procedure, assessment of the cylindrical refractive error was shown to lie within an intra-subject range of 0.15 D [24].

**Figure 2:**
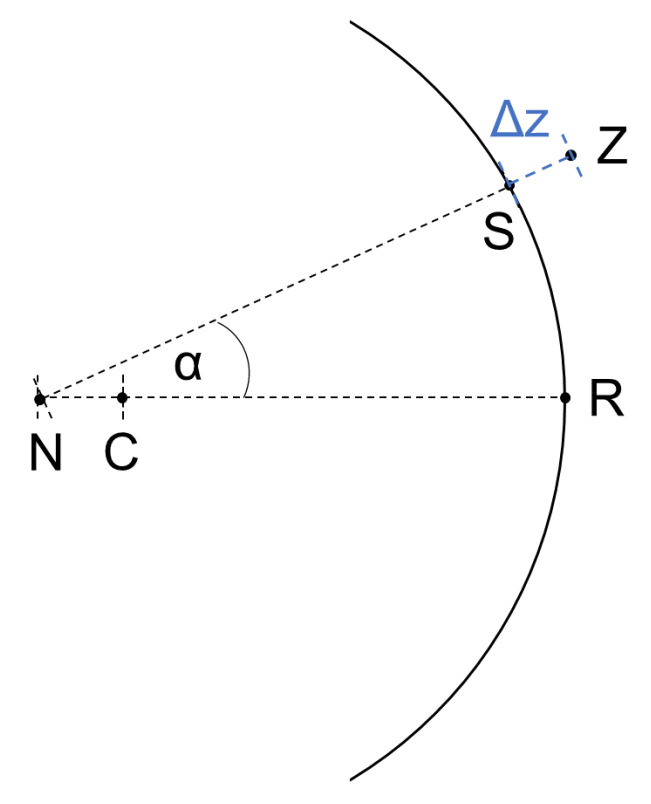
Process of retinal shape calculation from OCT imaging: scheme of an eye with the retinal curvature of the Arizona eye model [22], its circle center C, and ocular nodal point *N* (adjustable for the axial length). For each scan angle α, the peripheral refraction (PRX) shift Δz was added or subtracted for myopic or hyperopic defocus to the length $\bar{NS}$, respectively. A circle was then fitted to all resulting points *Z*. The image displays the case of myopic defocus and a subsequent elongation of $\bar{NS}$ by Δz.

Subsequently, the $0^{{}^{\circ}}$ and $90^{{}^{\circ}}$ meridian profiles were normalized to zero in order to obtain the relative PRX. Afterwards, they were size-adapted according to the participant’s axial length to match the angular size of the OCT line scan. The measured defocus in diopters for each eccentricity was then converted into millimeters by a factor of 0.32 mm per diopter, as derived from the optical properties of the Arizona eye model [22]. Subsequently, the reverse defocus shift Δz in millimeters was applied to the retina of the eye model with a baseline radius of 13.4 mm, as drawn schematically in Figure 2. The resulting shift of the baseline retina simulates the assumed retinal shape.

It is noteworthy that the eccentricity angles for the PRX measurements are relative to the axial-length-adjusted position of the nodal point *N* in the eye. However, this point does not coincidence with the circle center *C* of the baseline radius for the retina on the y-axis (see Figure 2). Therefore, the general circle equations needed to be adjusted in order to calculate the $x_{Z_{\left(\alpha \right)}}$ and $y_{Z_{\left(\alpha \right)}}$ positions of the points $Z_{\left(\alpha \right)}$ resulting from the PRX-defocus shift $\Delta z_{\left(\alpha \right)}$. This calculation was performed for each measurement angle α separately via Equation (1).

(1) $$\left(\begin{array}{c} x_{Z_{\left(\alpha \right)}}\\ y_{Z_{\left(\alpha \right)}} \end{array}\right)=\left(\begin{array}{c} 0\\ -\bar{CN} \end{array}\right)+\left({\bar{NS}}_{\left(\alpha \right)}-\Delta z_{\left(\alpha \right)}\right)\cdot \left(\begin{array}{c} \sin\alpha \\ \cos\alpha \end{array}\right)$$

Here, ${\bar{NS}}_{\left(\alpha \right)}$ represents the distance between the nodal point and the original eye model circle under a given angle αm and is derived from Equation (2).

(2) $${\bar{NS}}_{\left(\alpha \right)}=\bar{CN}\cdot \cos\alpha +\sqrt{{\bar{CN}}^{2}\cdot \cos^{2}\alpha -{\bar{CN}}^{2}+{\bar{CR}}^{2}}$$

As a last and equal step to the previously explained fitting procedure from OCT, a circle was fitted to the re-arranged points $Z_{\left(\alpha \right)}$. This radius was considered as the retinal radius of curvature, hereinafter called the “PRX-based retinal curvature”.

### 2.4. Statistical Data Analysis

Data analysis was carried out with the help of MATLAB (MATLAB 2020a, The MathWorks, Inc., Natick, MA, USA). Normal distribution was ensured with the Lilliefors test [25]. A *t*-test was performed in addition to Bland–Altman analysis [26] and the calculation of the intraclass correlation coefficient (ICC(2,1)) [27] in order to investigate the statistical differences and agreement between the OCT- and PRX-based retinal curvatures. Bivariate correlation analysis was performed with Pearson correlation [28]. Statistical results were interpreted as significant in the case that *p* < 0.05.

## 3. Results

The mean retinal radii from the OCT measurements and from the PRX meridians were as follows: 12.73 ± 1.05 mm (OCT), 13.63 ± 1.14 mm (PRX, $0^{{}^{\circ}}$ meridian), 12.82 ± 0.51 mm (PRX, $90^{{}^{\circ}}$ meridian), and 13.18 ± 0.52 mm (PRX, spherical equivalent).

OCT- and PRX-based retinal curvatures were compared for both meridians and the spherical equivalent values. The *t*-test, ICC, and Bland–Altman results are shown in Table 1. All investigated refraction profiles showed poor reliability for both measurement methods, with ICCs < 0.5. The *t*-tests exposed a significant difference between the OCT-based retinal curvature and PRX-based retinal curvature in the 0${}^{{}^{\circ}}$ meridian (*p* = 0.01). However, the differences of the spherical equivalent profile and the 90${}^{{}^{\circ}}$ meridian were only borderline significant and non-significant, respectively (*p* = 0.07 and *p* = 0.71). These findings fit to the Bland–Altman results.

**Figure 3:**
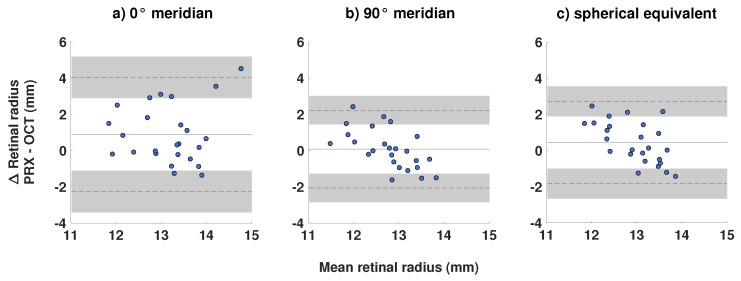
Bland–Altman plots for the comparison between PRX- and OCT-based retinal radii of curvature (*n* = 25) for the (**a**) 0${}^{{}^{\circ}}$ refractive meridian, (**b**) 90${}^{{}^{\circ}}$ refractive meridian, and (**c**) spherical equivalent refraction.

The Bland–Altman agreement revealed a positive mean difference in all meridians, meaning that the PRX-based retinal curvature was estimated to be larger than the OCT-based retinal curvature on average. The largest discrepancy was present for the 0${}^{{}^{\circ}}$ meridian (mean difference = +0.89 mm (−2.26 to +4.04 mm)), whereas the 90${}^{{}^{\circ}}$ meridian showed the best agreement (+0.08 mm (−2.07 to +2.36 mm)). As expected, the agreement for the spherical equivalent meridian was located in between (+0.45 mm (−1.83 to 2.72 mm)). The width of the limits of agreement was also largest for the 0${}^{{}^{\circ}}$ meridian, followed by the spherical equivalent and the 90${}^{{}^{\circ}}$ meridian with 6.30 mm, 4.55 mm, and 4.43 mm, respectively. Figure 3 visualizes the Bland–Altman analysis from Table 1. There was a notable trend for the 90${}^{{}^{\circ}}$ meridian and the spherical equivalent in Figure 3b,c. Here, the generally positive mean difference turned into a generally negative difference with increasing retinal radius of curvature.

**Figure 4:**
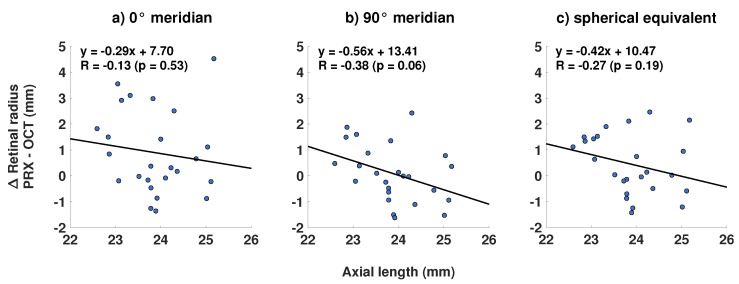
Linear regression equations and coefficients for the relation between axial length and the discrepancy of PRX- and OCT-based retinal radii of curvature (*n* = 25) for the (**a**) 0${}^{{}^{\circ}}$ refractive meridian, (**b**) 90${}^{{}^{\circ}}$ refractive meridian, and (**c**) spherical equivalent refraction.

As the OCT-based retinal radius increases with myopia [20], a correlation analysis between the radial difference and axial length was performed. The expected negative correlation between PRX- and OCT-based retinal curvature and axial length could be shown for all refractive meridians (see Figure 4). However, only the regression for the 90${}^{{}^{\circ}}$ meridian was borderline significant (R = −0.38; *p* = 0.06).

## 4. Discussion

The study compared PRX- and OCT-based retinal shapes with each other and in relation to axial length. It provided evidence that OCT-based and PRX-based retinal shapes differ from each other, revealing low ICCs. The PRX-based retinal radius was estimated to be larger compared to the OCT-based radius for all three investigated refractive profiles (0${}^{{}^{\circ}}$ and 90${}^{{}^{\circ}}$ meridians and spherical equivalent). The highest absolute deviations were revealed in the 0${}^{{}^{\circ}}$ meridian, and the lowest in the 90${}^{{}^{\circ}}$ meridian. This difference in retinal shape of 0.81 mm between meridians might arise from the increase of astigmatism in the peripheral retina, corresponding to approximately 2.5 D, as reported in [29,30,31]. Furthermore, this fact supports the hypothesis that direct methods of retinal shape estimation, such as OCT or magnetic resonance imaging (MRI), might lead to more reliable and stable results, as these are independent from eccentricity-related refractive fluctuations. In fact, absolute differences are low (on average, less than 1 mm), but more importantly, the discrepancy in the agreement is inconsistent across the range of evaluated retinal curvatures and axial lengths. With increasing axial length, the Δ(PRX—OCT) difference of radii decreased, and eventually even became negative. This tendency could be interpreted as an overestimation of PRX-based retinal steepness with increasing axial length and, therefore, myopia. Vice versa, it could be described as an overestimation of PRX-based retinal flatness with decreasing axial length and, therefore, emmetropia or hyperopia. This effect could be explained by the influence of higher-order aberrations occurring with off-axis refraction. Higher-order aberrations alone sum up to 0.9 D in the periphery (assuming a 4 mm pupil) [32] and, therefore, can change measurements from refraction when only lower-order aberrations are considered. As recently found, PRX profiles in emmetropic children show much flatter courses with less relative peripheral myopia and inter-subject variability when measured with an open-field wavefront aberrometer over ±30${}^{{}^{\circ}}$ of visual field [33]. Using the same wavefront aberrometer technique, myopes also show flatter profiles with less relative peripheral hyperopia than usually reported [34]. These findings would, in turn, lead to larger PRX-based retinal curvatures in myopia and smaller retinal curvatures in emmetropia, and are thus a better match with the OCT-based retinal shapes.

When comparing these results to previous literature, there are inconsistent findings due to methodological differences. The most important study to recognize in this context is by Verkicharla et al. [35]. They compared optical biometry and Dunne’s method [36,37] (Gullstrand eye model based on PRX modeling) for retinal shape estimations, and compared them to the ground truth of magnetic resonance imaging (MRI). Dunne’s method was less appropriate than optical biometry and showed flatter retinal shapes of 1.7 mm compared to MRI contours. In this sense, the aforementioned study agrees that PRX is not ideally suited for estimating retinal contour. The smaller mean difference in the current study most likely arises from the usage of the same eye model for OCT- and PRX-based retinal shape estimations. It is further noteworthy that the present study considered the OCT-based retinal shape as the ground truth instead of MRI. Nevertheless, a good agreement between the retinal shapes measured via MRI and distortion-corrected OCT scans was found previously [38]. OCT also provides a faster, more clinically feasible, and less expensive method for retinal imaging than MRI. With a somewhat different methodology, but with a similar purpose to that of the present study, Schmid [16] found that relative peripheral hyperopia predicts more retinal steepness in children, but that there is considerably high inter-subject variability in both parameters. Similar results were found later in adults [19]. However, both studies used an open-field autorefraction and optical biometry with off-axis fixation via eye turns, as opposed to the eccentric photorefraction and OCT measurements with constant primary gaze position in the current study.

The strengths of this current study are laid down in the methodology. A fast eccentric photorefractor [23] was used, which abolishes the need for eye movements for peripheral fixation targets. The same advantage accounts for the OCT measurements vs. optical biometry. Moreover, the sampling rate was higher for both parameters of interest. For PRX, measurements were performed in steps of 0.71${}^{{}^{\circ}}$ instead of 5${}^{{}^{\circ}}$ to 10${}^{{}^{\circ}}$ steps, as with common open-field autorefractors. With regards to OCT, each 11th A-scan out of a total of 1024 A-scans was used for the retinal shape estimation, which corresponds to a sampling rate of approximately 0.6${}^{{}^{\circ}}$. However, there are also certain limitations present with the current study. Despite consisting of myopes and emmetropes, the sample lacked highly myopic participants with an axial length of >26 mm, which could have led to more pronounced and significant differences and correlation results. Moreover, the prevalence of staphyloma [39] increases with higher degrees of myopia, leading to more irregular retinal shapes and PRX profiles, as well as an unknown relationship between them. The eye model was adjusted for the individual axial length, but not for other optical components, such as corneal curvature. However, tolerance analysis showed that axial length appears to be the largest error source for retinal shape calculation (up to ±4 mm misestimation), while corneal curvature or lens refractive index account for only up to ±0.2 and ±0.1 mm of misjudgment [21]. The Arizona eye model [22] was figured as the baseline for the conversion from diopters to millimeters for the PRX-assumed retinal shape. However, this conversion factor was not adjusted for the individual participants with their refractive ocular properties, but was rather inferred from the eye model. This could lead to inaccuracies, which are pronounced in the peripheral measurement angles. Moreover, only the horizontal visual field was investigated in the current study due to the technical set-up of the photorefractor, which did not allow conclusions for the vertical visual field. The first rough suggestions match in the sense that PRX profiles [40], as well as retinal curvature [20], have been shown in separate studies to be less affected by myopia in the vertical visual field than horizontal visual field.

The current study has shown that retinal shape should be preferably inferred from direct imaging methods, such as OCT instead of an indirect estimation from PRX profiles. However, the measurements of PRX should not be neglected at all, since it is of very high importance especially in the field of myopia control. In contrast to retinal shape (which figures more as a biomarker still), PRX can be altered in a clinically feasible way using optical treatment strategies, such as orthokeratology [41,42], progressive addition spectacle lenses [43], multifocal contact lenses [44] or refractive surgery [45]. The overall aim of these options is to shift PRX into a myopic direction, where orthokeratology lenses show a particular strong effect mainly outside of 15° eccentricity in four investigated directions [41]. Therefore, it is recommended that future studies and clinical applications should strive for an evaluation of PRX and retinal shape as separately measured parameters, albeit brought into a meaningful combination.

From the study results, it can be concluded that retinal shape measurements from PRX measurements lead to overall larger retinal radii compared to radii derived from distortion-corrected OCT imaging. However, this relationship is reversed with increasing axial length, where PRX tends to underestimate the radius of the retina when compared to OCT. Therefore, future studies should rely more on direct retinal shape estimations from OCT rather than indirectly inferring it from PRX measurements, especially in the context of myopia.

## Figures and Tables

**Table 1:** *t*-tests, intraclass correlation coefficients (ICCs), and Bland–Altman analysis of the PRX-based retinal curvature for three refractive profiles versus the OCT-based retinal curvature (*n* = 25).

	0${}^{{}^{\circ}}$ Meridian	90${}^{{}^{\circ}}$ Meridian	Spherical Equivalent
**ICC analysis**			
ICC	0.18	0.20	0.03
*p*-value	0.65	0.29	0.47
**Bland-Altman analysis (mm)**			
Mean difference	+0.89	+0.08	+0.45
Upper limit of agreement (95% confidence interval)	+4.04 (+2.89 to +5.19)	+2.36 (+1.45 to 3.02)	+2.72 (+1.89 to +3.55)
Lower limit of agreement (95% confidence interval)	−2.26 (−1.11 to −3.41)	−2.07 (−1.28 to −2.85)	−1.83 (−1.00 to −2.66)
***t*** **-test**			
H${}_{0}$/H${}_{1}$	H${}_{1}$	H${}_{0}$	H${}_{0}$
*p*-value	0.01	0.07	0.71

## Data Availability

Not applicable.

## Abbreviations

The following abbreviations are used in this manuscript:

OCT	Optical coherence tomography
RPE	Retinal pigment epithelium
PRX	Peripheral refraction
ICC	Intraclass correlation coefficient
MRI	Magnet resonance imaging
